# Concentrations of canine prostate specific esterase, CPSE, at baseline are associated with the relative size of the prostate at three-year follow-up

**DOI:** 10.1186/s12917-021-02874-1

**Published:** 2021-04-26

**Authors:** Bodil S. Holst, Sofia Carlin, Virginie Fouriez-Lablée, Sofia Hanås, Sofie Ödling, Liss-Marie Langborg, S. J. Kumari A. Ubhayasekera, Jonas Bergquist, Jesper Rydén, Elin Holmroos, Kerstin Hansson

**Affiliations:** 1 Department of Clinical Sciences, Swedish University of Agricultural Sciences, Box 7054, SE-750 07 Uppsala, Sweden; 2 Diagnostic Imaging Clinic, University Animal Hospital, Swedish University of Agricultural Sciences, Uppsala, Sweden; 3 Evidensia Specialist Animal Hospital Strömsholm, Strömsholm, Sweden; 4 ReDog Veterinary Clinic, Västerås, Sweden; 5 Department of Chemistry – Biomedical Center, Analytical Chemistry, Uppsala University, Uppsala, Sweden; 6 Department of Energy and Technology, Applied Statistics and Mathematics, Swedish University of Agricultural Sciences, Uppsala, Sweden

**Keywords:** Dog, Steroids, Corticosteroids, Prostate hyperplasia, Biomarker, Ultrasound

## Abstract

**Background:**

Enlargement of the prostate is associated with prostatic diseases in dogs, and an estimation of prostatic size is a central part in the diagnostic workup. Ultrasonography is often the method of choice, but biomarkers constitute an alternative. Canine prostate specific esterase (CPSE) shares many characteristics with human prostate specific antigen (PSA) and is related to prostate size. In men with clinical symptoms of prostatic disease, PSA concentrations are related to prostate growth. The aims of the present follow-up study were to evaluate if the concentration of CPSE is associated with future growth of the prostate, and if analysis of a panel of 16 steroids gives further information on prostatic growth. Owners of dogs included in a previous study were 3 years later contacted for a follow-up study that included an interview and a clinical examination. The prostate was examined by ultrasonography. Serum concentrations of CPSE were measured, as was a panel of steroids.

**Results:**

Of the 79 dogs included at baseline, owners of 77 dogs (97%) were reached for an interview, and 22 were available for a follow-up examination. Six of the 79 dogs had clinical signs of prostatic disease at baseline, and eight of the remaining 73 dogs (11%) developed clinical signs between baseline and follow-up, information was lacking for two dogs. Development of clinical signs was significantly more common in dogs with a relative prostate size of ≥2.5 at baseline (*n* = 20) than in dogs with smaller prostates (*n* = 51). Serum concentrations of CPSE at baseline were not associated with the change in prostatic size between baseline and follow-up. Serum concentrations of CPSE at baseline and at follow-up were positively associated with the relative prostatic size (S_rel_) at follow-up. Concentrations of corticosterone (*P* = 0.024), and the class corticosteroids (*P *= 0.0035) were positively associated with the difference in S_rel_ between baseline and follow-up.

**Conclusions:**

The results support the use of CPSE for estimating present and future prostatic size in dogs ≥4 years, and the clinical usefulness of prostatic size for predicting development of clinical signs of prostatic disease in the dog. The association between corticosteroids and prostate growth warrants further investigation.

**Supplementary Information:**

The online version contains supplementary material available at 10.1186/s12917-021-02874-1.

## Background

The prostate is an androgen-dependent gland. In intact middle-aged and old dogs, an enlarged prostate is a natural sequel to androgen production, and with age almost all will develop benign prostatic hyperplasia (BPH) [1–3]. The type of hyperplasia varies with age, from a glandular hyperplasia in younger dogs to a complex form with glandular hyperplasia, increased amount of stroma, often cystic alveoli and chronic inflammation in older dogs [1, 2]. The prostate growth is more pronounced during the first 4 years and then reaches a plateau [2]. After 4 years, a correlation between the size and age is not always evident [4], and in senior dogs, an involution may occur [5]. Although growth is androgen dependent, it is not related to increasing testosterone concentrations. Instead, a mild decrease in serum testosterone concentrations is seen with increasing age, while concentrations of estrogens remain similar or increase [3, 6]. As a consequence, the testosterone/estradiol relationship has been described as a measurable indicator of BPH [7]. In men, both estrogen receptor (ER) α and β have been described to be more strongly expressed in epithelial cells of hyperplastic than of normal prostates [8]. Beside a decreased androgen/estrogen ratio, several other factors, including stromal growth factors and chronic inflammation, have been indicated to play a role in the pathogenesis of BPH [9].

Although most, if not all, intact males will develop BPH with time, only a sub-population will show related clinical signs, often associated with a prostate that is markedly increased in size [10]. Signs related to BPH include ribbon-like stools [11], hematuria, incontinence and tenesmus [12]. The sperm quality may be affected leading to fertility problems [13]. Clinical signs may be insidious in nature, and, at least initially, go unnoticed by owners [4]. The fact that many male dogs have subtle signs of BPH, and veterinary care may not be sought until the signs have become severe, makes BPH a potential welfare problem in the ageing male. In addition, presence of BPH increases the risk of developing other prostate diseases, mainly prostatitis, a condition that may be life-threatening [12].

Because BPH and most other prostatic diseases are associated with an enlargement of the prostate, estimation of prostatic size is a central part in the diagnostic workup. Rectal palpation is a quick and easy first step [14], although not always feasible in larger dogs, or in older dogs, as the position of the prostate often changes with age from the pelvic to the abdominal cavity, and ultrasonography is often the method of choice [15, 16]. An ultrasound examination not only gives information on size, but also on shape, contour, echogenicity and symmetry, and it also adds information on adjacent soft tissue [17]. The drawback is related to availability of equipment and skill of the veterinarian performing the investigation. An alternative for estimating an increased prostatic size is analysis of serum concentration of the biomarker canine prostate specific esterase (CPSE) [4]. CPSE is an arginine esterase, and the major secretory product of the canine prostate [18]. Serum CPSE concentrations are significantly higher in dogs with BPH than in normal dogs [7, 19, 20]. Several authors have suggested inclusion of CPSE analysis in the diagnostic workup of dogs with clinical signs of BPH, and for screening of geriatric dogs [4, 21, 22]. There are several similarities between CPSE in dogs and prostate specific antigen (PSA) in men. Both are serine proteases with similar regulation [18, 23]. In men, it has been described that the concentrations of PSA may predict the future growth of the prostate gland [24]. If this would be the case also for CPSE in dogs, the diagnostic value of a CPSE analysis would increase further.

The aims of the present follow-up study were to evaluate if the concentration of CPSE is associated with future growth of the prostate, and if analysis of a panel of 16 steroids gives further information on prostatic growth.

## Results

**Fig. 1:**
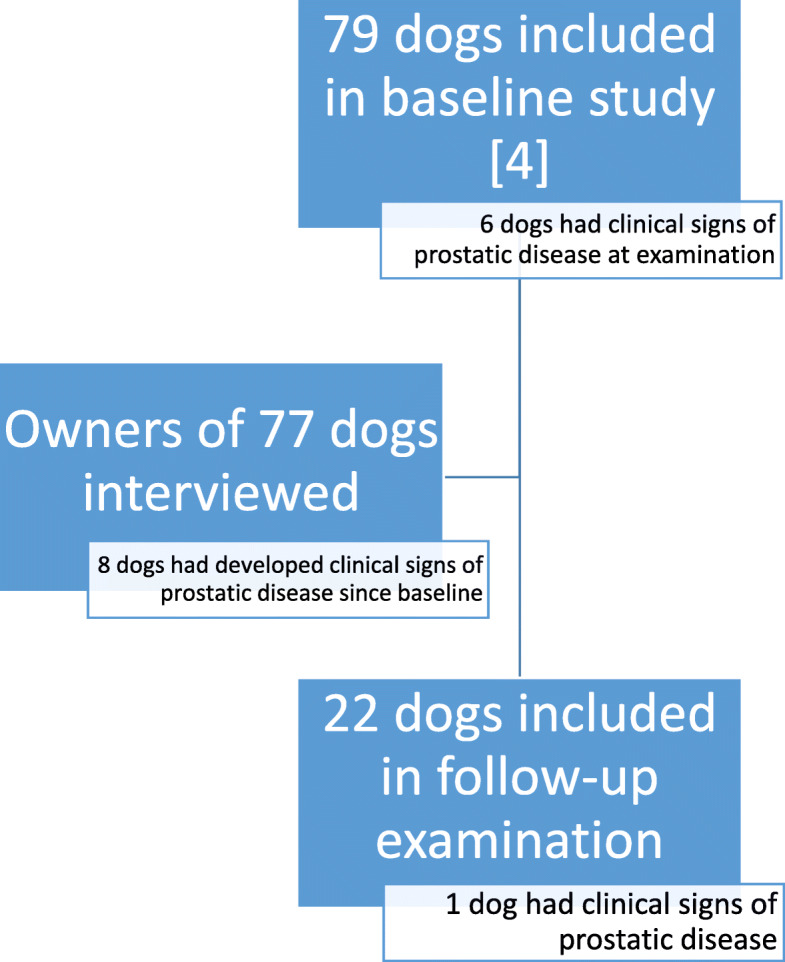
Chart of the study design with number of dogs included in the different steps

The number of dogs included in the different parts of the study is summarized in Fig. 1.

### Interview

Of 79 dogs included at baseline, six dogs had clinical signs of prostatic disease and 73 dogs had no such clinical signs. Contact was established with owners of 77 dogs (97%). The interviews revealed that in addition to the six dogs with clinical signs at inclusion, eight dogs (8/73, 11%) had developed clinical signs of prostatic disease since baseline, the most common being haematuria, urinary incontinence and tenesmus. The median baseline concentration of CPSE for dogs that later developed clinical signs was 135 ng/ml (interquartile range, IQR 71–402 ng/ml), and for dogs that did not develop clinical signs 61 ng/ml (IQR < 25 to 130 ng/ml). The difference between these groups was not significant (*P* = 0.1). Dogs with a S_rel_ of ≥2.5 at baseline (*n* = 20) had significantly more often than those with a smaller S_rel_ (*n* = 51) developed clinical signs of prostatic disease (*P* = 0.035). Information from two dogs was lacking. Median (IQR) S_rel_ at baseline was 3.0 (1.7–3.5) for dogs that had developed clinical signs and 1.8 (1.3–2.4) for dogs that had not developed clinical signs.

Of the 79 dogs that were included at baseline, 22 (28%) were available for a follow-up examination. Reasons for not participating in the follow-up were for the other 57 dogs euthanasia (*n* = 25), surgical castration (*n* = 8), treatment with osaterone acetate (*n* = 7), long distance (*n* = 7), treatment with deslorelin (*n* = 6), no answer (*n* = 2), treatment with both osaterone acetate and deslorelin (*n* = 1), or was not interested in participating (*n* = 1). The most common causes for euthanasia were neoplasia (*n* = 11), multifactorial (*n* = 5), age (*n* = 4), and joint problems (*n* = 4).

### Ultrasonography, CPSE and steroid concentration

Of the 22 dogs available for follow-up, 21 did not have signs of prostatic disease. One Rottweiler had a sanguineous urethral discharge that was evident when in contact with bitches in heat. The S_rel_ of his prostate was 2.9, and had not changed significantly since baseline. He had > 5 intra-prostatic cysts at follow-up.

The 22 dogs were of 14 different breeds. Their median age and weight at baseline were 5.2 years (IQR 4.0–7.0) and 27.8 kg (IQR 12.5–37.5), respectively. At follow-up, their median age was 8.8 years (IQR 7–10.3) and median weight was 26.3 kg (IQR 13.8–37.7). At baseline, 27% of these 22 dogs had prostates with a relative size ≥2.5, and at follow-up this proportion was 41%.

**Fig. 2:**
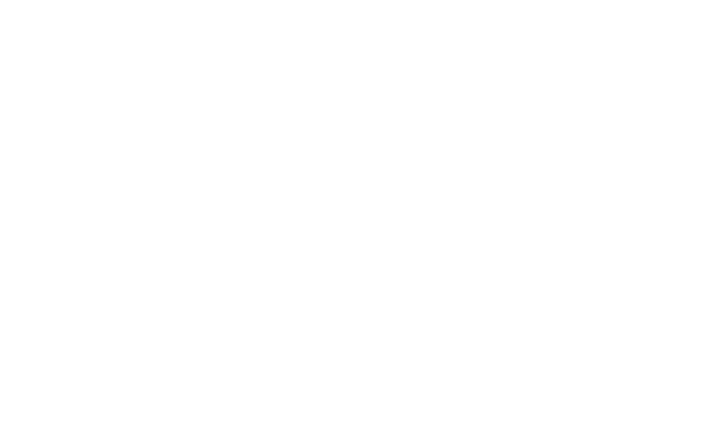
Change in relative size, S_rel_, of the prostate between baseline and follow-up, in dogs (*n* = 22) of various ages

The V and S_rel_ had increased slightly but significantly from baseline to follow-up (*P* = 0.006, Table 1). The volume had increased in 12 dogs (median age 9.5 years (IQR 8.0–11.8), median CPSE concentration at baseline 59.1 ng/ml (IQR 15.4–111.0)). For nine dogs, volume had changed less than 15%. Their median age was 7 years (IQR 7.0–8.8), median CPSE concentration at baseline 47 ng/ml (28.1–77.0). The volume of the prostate had decreased 27% in one 13-year-old dog with CPSE at baseline 95 ng/ml. The change in S_rel_ between baseline and follow-up in individual dogs is shown in Fig. 2.

**Table 1:** Serum concentration of CPSE, size of the prostate, and dogs with presence of cysts at baseline and follow-up (*n* = 22)

Parameter		Baseline	Follow-up	*P*-value
CPSE, ng/ml	Median (IQR)	52 (24–97)	107 (49–416)	0.001
V, cm^3^	Median (IQR)	18 (12–30)	21 (15–38)	0.006
S_rel_	Median (IQR)	1.8 (1.2–2.2)	2.2 (1.4–2.9)	0.006
Cysts, presence	N (%)	12 (54)	18 (82)	0.18

*CPSE* canine prostate specific esterase, *V* Volume of the prostate, *S*_*rel*_ relative size of the prostate, *Cysts* prostatic cysts

Using multiple regression, baseline CPSE concentrations and concentrations at follow-up were significantly associated with S_rel_ at follow-up (Table 2). The Pearson correlation coefficient (*r*) between baseline CPSE and Srel at follow up was 0.53 (*P* = 0.011). Age and weight were not significantly associated with S_rel_ at follow-up. None of the steroids, analyzed separately, as classes or as ratios, were significantly associated with the S_rel_ at follow-up. One steroid, corticosterone was associated with the difference in S_rel_ between baseline and follow-up (regression coefficient 0.5, adjusted *R*^*2*^ = 0.75, *P* = 0.024), as was the class corticosteroids (regression coefficient 0.008, adjusted *R*^*2*^ = 0.34, *P* = 0.003). Median concentrations of steroids in dogs with different S_rel_ are presented in Table 3.

**Table 2:** Association between CPSE concentration at baseline and follow-up, and relative prostatic size at follow-up, adjusted *R*^*2*^ = 0.51

Parameter	Regression coefficient	Standard Error	t-value	*P*-value
CPSE baseline	0.005	0.0016	3.3	0.004
CPSE follow-up	0.003	0.00079	3.5	0.003

*CPSE* canine prostate specific esterase

**Table 3:** Concentration of the different steroids, classes of steroids, ratios of classes of steroids and of canine prostate specific esterase (CPSE) in dogs at follow-up

Steroid	Prostatic size	N	Median (ng/ml)	IQR
Aldosterone	< 2.5	13	0.10	0.04–0.31
	≥2.5	9	0.26	0.17–0.44
Androsterone	< 2.5	13	0.069	0.04–0.16
	≥2.5	9	0.041	0.01–0.15
Androstenedione	< 2.5	13	1.43	0.4–2.16
	≥2.5	9	2.20	1.2–3.4
Cortisol	< 2.5	13	36.7	17.3–58.6
	≥2.5	9	21.5	14.5–38.9
Cortisone	< 2.5	13	14.75	10.4–20.32
	≥2.5	9	10.41	7.5–23.60
Corticosterone	< 2.5	13	1.81	1.2–3.77
	≥2.5	9	1.38	0.7–2.61
11-Deoxycorticosterone	< 2.5	13	0.40	0.13–0.79
	≥2.5	9	0.17	0.076–0.31
11-Deoxycortisol	< 2.5	13	6.82	4.2–8.39
	≥2.5	9	4.86	1.96–8.29
DHEA	< 2.5	13	0.63	0.34–2.53
	≥2.5	9	1.56	0.58–2.32
Etiocholanolone	< 2.5	13	0.24	0.18–0.32
	≥2.5	9	0.34	0.29–0.44
17α-hydroxyprogesterone	< 2.5	13	0.11	0.079–0.22
	≥2.5	9	0.14	0.070–0.21
Pregnanolone	< 2.5	13	0.46	0.34–0.60
	≥2.5	9	0.46	0.39–0.54
Progesterone	< 2.5	13	0.14	0.078–0.29
	≥2.5	9	0.14	0.065–0.22
Testosterone	< 2.5	13	1.40	0.98–3.30
	≥2.5	9	2.41	2.17–4.59
Estrone	< 2.5	13	0.023	0.0004–0.05
	≥2.5	9	0.036	0.013–0.059
Estradiol	< 2.5	13	0.0072	0.00–0.017
	≥2.5	9	0.0075	0.00–0.016
CPSE (follow-up)	< 2.5	13	83.0	29.6–118.4
	≥2.5	9	153.3	91.8–550.2
SUM estrogens	< 2.5	13	0.039	0.0069–0.059
	≥2.5	9	0.036	0.021–0.078
SUM androgens	< 2.5	13	3.45	2.11–8.5
	≥2.5	9	9.13	4.45–11.6
SUM corticosteroids	< 2.5	13	57.43	43.36–91.21
	≥2.5	9	39.6	28.3–71.0
SUM progestins	< 2.5	13	0.79	0.602–1.01
	≥2.5	9	0.69	0.625–1.074
Androgens/estrogens	< 2.5	11	140	87–301
	≥2.5	9	202.5	96.2–481.9
Progestins/estrogens	< 2.5	11	23.4	11.6–31.6
	≥2.5	9	23.05	9.64–30.66
Corticosteroids/estrogens	< 2.5	11	1838	619–3710
	≥2.5	9	1127	392–5148
Androgens/progestins	< 2.5	13	5.44	2.5–11.27
	≥2.5	9	8.34	7.27–16.47
Corticosteroids/androgens	< 2.5	13	19.18	6.44–28.19
	≥2.5	9	5.66	3.33–10.50
Corticosteroids/progestins	< 2.5	13	84.6	44.6–104.5
	≥2.5	9	63.1	40.2–100.3

The shape of the prostate was symmetrical both at baseline and at follow-up in 17 dogs. It changed from asymmetrical to symmetrical in two dogs and from symmetrical to asymmetrical in three dogs. The contour remained smooth in all dogs. The parenchyma did not change for 17 dogs; seven with a homogenous and 10 with heterogeneous echogenicity. It changed to heterogeneous in four dogs and to homogeneous in one dog. At baseline, 12 dogs (54%) had prostatic cysts, all with one to five cysts. At follow-up, 18 dogs (82%) had detectable cysts: five had one to five cysts as previously, and five had an increased number of cysts (> 5) compared to baseline. Of 10 dogs that did not have any cysts at baseline, eight had developed one to five cysts at follow-up.

## Discussion

Circulating biomarkers for the evaluation of the canine prostate are valuable for the practicing veterinarian with limited access to an ultrasound equipment. Concentrations of CPSE are related to the size of the prostate [4] and to the presence of BPH [7, 19, 20]. The analysis of CPSE can therefore contribute with important information both in the workup of cases with clinical signs related to the prostate, and in the screening panel for old male dogs. The samples must be taken after a proper sexual rest, as ejaculation increases the CPSE concentration in serum [25]. We have previously described that clinical signs were more common in dogs with a S_rel_ of ≥2.5, with a CPSE concentration of ≥90 ng/ml [4]. In the present study, dogs with a baseline S_rel_ ≥ 2.5 significantly more often had developed clinical signs of prostatic disease at follow-up than those with smaller S_rel_. This supports the clinical usefulness of this cut-off. The concentrations of CPSE did not differ between dogs that had developed clinical signs and those that had not done so. We have previously described an association between CPSE concentrations and size of the prostate [4], and the lack of association between CPSE concentrations and development of clinical signs in the present study may be due to the relatively small study population, with eight of 73 dogs developing clinical signs.

Concentrations of PSA in men with moderate to severe clinical signs and an enlarged prostate have been described to predict prostate growth [24]. The PSA analysis thus contributes with specific information in this group of patients. In the present study, CPSE concentrations at baseline were significantly correlated with S_rel_ at follow up. The concentration of CPSE is thus not only correlated with present prostatic size, but also with the future size. However, although CPSE is similar to PSA, there was no association between baseline CPSE concentrations and the difference in S_rel_ between baseline and follow-up. The study population of dogs differed from the study in men in that most dogs were without clinical signs, and not all had enlarged prostates. It may therefore be that an association between CPSE and difference in S_rel_ would have been detected with a different study population, including only dogs with enlarged prostates and clinical signs, or with a larger study population.

In the present study, 11% of the dogs from the baseline study with owners that were interviewed had, according to their owners, developed clinical signs of prostatic disease during the 3-year-period after the examination at baseline. These results highlight that clinical signs related to the prostate are common in intact male dogs > 4 years. When included in the study at baseline, these dog owners became aware of prostatic diseases, and this awareness, together with the fact that all dogs were > 4 years old, may have contributed to this relatively high proportion of dogs with clinical signs.

In a previous study on dogs registered in a teaching veterinary hospital database, 0.7% had prostatic disorders [26]. This much lower number is likely influenced by the age distribution. It has previously been described that a large proportion of visits to an animal hospital consists of young dogs [27]. Another contributing factor is likely a proportion of castrated dogs, and another breed distribution, as the prevalence of prostatic disease differs between different breeds and sizes of dogs [26]. Clinical signs of prostatic disease may be insidious and may not lead to a visit at a veterinarian [4]. A low awareness of prostatic disease among general dog owners indicates that dogs getting veterinary care for this condition are only the tip of an iceberg. Due to this, many male dogs may suffer unnecessarily from a curable condition. Prevention screening of the prostate of male dogs has been recommended [4, 28], and is supported by the present study. The present results also point to the education of dog owners on the clinical signs of prostate disease, and possibilities for treatment, as a means to increase health and welfare of male dogs.

Despite the relationship between BPH and size of the prostate, and between prostate size and clinical signs, it should be noted that although 41% of the dogs at follow-up had S_rel_ > 2.5, only one of them (4%) had clinical signs of prostatic disease. In men, prostate size correlates poorly with clinical signs [29], indicating that other factors contribute. One such factor is prostatic fibrosis that is related to clinical signs in men [30]. Collagen structure in prostates of dogs and men is similar [31]. The majority of collagen is localized to the prostatic urethra, and a dense collagen network is present in capsular regions. Older, intact dogs have denser collagen fibers and thicker capsular fibers than young, intact males [31]. The presence of urinary signs in male dogs may thus be related to fibrosis, as in men. In men, the prostate has different zones, with BPH occurring more commonly in the transitional zone, surrounding the urethra [32], which is one reason for signs not being directly related to the overall size of the prostate. The canine prostate does not have different zones, and glandular proliferation occurs in all portions of the gland [1, 15]. However, more nodular changes may be present, and cysts more common, in the periurethral area [1]. In older dogs, when the hyperplasia commonly is of the complex type, the presence of cysts in this area may be related to blood-tinged discharges.

During the 3 years between baseline and follow-up, S_rel_ of the prostate had increased significantly. This is in accordance with previous studies showing that the prostate gland increases in size with age in intact males [1, 2]. However, the median increase was rather low. The same pattern was not seen in all dogs, and in 45% of the dogs, the prostate had remained similar in size, or even decreased. The decrease in S_rel_ in one dog may be caused by senile involution [5] and the proportion with similar S_rel_ likely represents the plateau after 4 years, described previously [2]. Not only the S_rel_ increased between baseline and follow-up, so did the proportion of dogs with cysts, although not significantly. At baseline all dogs with cysts had only few cysts, but at follow-up five of the 18 dogs with cysts had more than five cysts. The shape of the prostate was symmetrical both at baseline and at follow-up in 77% of the dogs. The presence of prostatic cysts is typical for the complex form of BPH, dominating from 7 years of age in beagles [1].

The pathogenesis behind BPH is complex, and modifiable as well as non-modifiable risk factors are suggested in men, including inflammation, growth factors and endocrine factors [9, 33]. A role for steroids in the development of BPH has been indicated since long. Acinar basal cells in the canine prostate are the major proliferative cell type, and express the androgen receptor [34], and BPH is an androgen-dependent condition. However, although the prevalence of BPH increases with age, testosterone concentrations tend to decrease [3]. In the present study, we used SFC-MS/MS to evaluate the concentration of 16 steroids in relation to the size of the prostate. The advantage with this method is the possibility to identify and quantify a large number of steroids within a short time using a small sample volume. Panels of steroids, and ratios between them, have been described to potentially have advantages in personalized medicine for men with prostatic cancer [35]. In the present study, no single steroid, class of steroids or ratio between different classes were associated with S_rel_ at follow-up. The disadvantage of this study is the relatively low number of dogs included in this analysis, leading to reduced power, which may be one explanation for this lack of significant associations. Corticosterone and the class corticosteroids were positively associated with the difference in S_rel_ between baseline and follow-up. The association between the difference in S_rel_ and corticoids is interesting. Chronic stress activates the hypothalamic-pituitary-adrenal axis, leading to increased concentrations of corticosteroids and inflammatory responses, and inflammation has been suggested as a contributing factor to BPH [33]. However, corticosterone is not a primary corticosteroid in dogs, and the results should therefore be interpreted with caution. Further studies are required before any conclusions can be drawn on the relationship between stress and BPH in dogs.

Although owners of 97% of the dogs that were included at baseline were reached for interviews, only 28% of the dogs were available for a clinical follow-up. A general problem with follow-up studies of conditions affecting middle-aged or older dogs is that a proportion of them will be euthanized or treated so that they have to be excluded. In the present study, 32% had been euthanized, many from tumours or joint disease, previously described common causes of mortality [36]. Twenty-eight per cent of the dogs included at baseline were excluded because of the exclusion criteria; surgical or medical castration, or treatment for prostatic disease. Only one dog owner expressed no interest in participating in the clinical follow-up. The included dogs can therefore be considered representative for intact male dogs that have not been treated for prostatic disease.

## Conclusion

CPSE concentrations at baseline and follow-up are significantly associated with S_rel_ at follow-up but not with the difference in S_rel_ between baseline and follow-up. Corticosteroids, and specifically corticosterone, were associated with the difference in S_rel_ between baseline and follow-up. Dogs with a baseline S_rel_ ≥ 2.5 were significantly more likely to develop clinical signs of prostatic disease than other dogs. Clinical signs had developed in 11% of dogs > 4 years old during the three-year follow-up period.

## Methods

### Study design

Owners to dogs included in a previous study [4] were contacted and asked if they were willing to participate in a follow-up. The follow-up included an interview and a clinical study. In the clinical study, breed, age, and weight were recorded, as was information on clinical signs of possible prostatic disease. Ultrasonography of the prostate was performed at one of the two animal hospitals and two animal clinics that participated in the original study. A venous blood sample was collected for analysis of CPSE and steroid hormones.

### Interview

Owners were contacted via phone (Additional file 1). Questions included if the dog was still alive, and if it was or had been treated for prostatic problems. The owners were also asked if the dog had been surgically castrated or if it was treated with deslorelin. If the dog was not alive, the owner was questioned about the reason for euthanasia.

### Inclusion and exclusion criteria for clinical part

Inclusion criteria were male dogs that had been included in the previous study (baseline) [4]. Exclusion criteria were treatment for prostatic disease or castrated since baseline (3 years earlier).

### Ultrasonography

The prostate was examined using the previously described standardized protocol [4] by experienced veterinary surgeons that had adequate equipment^1^. Transducer type and frequency were optimized for the individual dog to cover the entire prostate for measurements and to get as good detail resolution as possible for the parenchymal evaluation.

Length, width, and height of the prostate were measured three times on different still images and the mean values were used in further calculations. The prostatic volume (V) for the individual dog was calculated according to Kamolpatana et al. as V = (1/2.6) * (Length * Width * Height) + 1.8 cm^3^ [16]. In addition prostatic parenchyma (homogenous or heterogenous), contour (smooth or uneven); shape (symmetrical or asymmetrical), and presence of cysts (none, 1–5 or > 5) were recorded.

### Analysis of CPSE

Venous blood samples were collected and serum was stored at − 70 °C until CPSE was analyzed using a commercial ELISA; Odelis® CPSE (Virbac). Intra assay coefficient of variation (CV) was < 5% and interassay CV was < 7%, according to the manufacturer. Samples with concentrations ≥200 ng/mL were diluted 1:10 and re-analyzed.

### Analysis of steroid hormones

An analysis of steroid hormones was performed by supercritical fluid chromatography–tandem mass spectrometry (SFC–MS/MS, Waters Corporation, Milford, MA, USA) system [37]. A slightly modified method of liquid -liquid extraction was used [37]. In brief, a 50 μL of plasma was spiked with 50 μL mixture of a corresponding deuterated internal standard mixture (10 μg/mL). The protein precipitation was achieved by the addition of 100 μL of ice cooled MeOH and followed by the addition of 200 μL of MTBE, then vortexed for 15 min. Samples were centrifuged at 13,000 rpm for 10 min at 4 °C. The supernatant was transferred to a clean tube and evaporated under a stream of nitrogen and the steroids were derivatized in to their methoximes prior to analysis [37]. The SFC system was equipped with a Acquity UPC^2^ BEH column (150 mm × 3.0 mm, 1.7 μm particle size; Waters, Milford, MA, USA). It was kept at 40 °C and at a mobile phase flow rate of 2 mL/min. The gradient program was started with 98% A (CO_2_, 99.999%) and 2% B (0.1% formic acid in methanol/isopropanol (1:1)), linearly increased to 17% B over 3 min, held at 17% B for 0.5 min, followed by a linear gradient down to 2% B over 0.5 min. Finally, it was held for 1 min at 2% B for the elution of ionic liquids out of the instrument, resulting in a total separation time of 5 min. Mass spectrometric detection was performed using electrospray ionization in the positive ionization mode (ESI+) with a capillary voltage of 2.8 kV, cone voltage of 30 V, and source offset of 30 V. Nitrogen and argon (0.15 mL/min) served as the desolvation gas and the collision gas, respectively. Desolvation temperature was maintained at 500 °C. The quantification of the analytes was based on a multiple reaction monitoring (MRM) coupled to stable isotope dilution. The limit of quantification (LOQ) and co-efficient of variation of steroids were 0.1–0.5 ng/mL and 8–10%, respectively. The recovery of steroids was more than 85%. Linear rage of the quantification for steroids was 0.1–1000 ng/mL. All data was acquired, analysed and processed using the Waters MassLynx software (version 4.1, Waters, Milford, MA, USA).

### Statistical analysis

The normal prostatic volume (V_norm_) was calculated according to Sannamwong and coworkers [38]. The formula includes weight of the dogs, and is based on healthy dogs aged 1.5–4 years: V_norm_ = 0.33 * BW (kg) + 3.28. The calculated volume (V) was compared to V_norm_ to get a relative value of the prostatic size: S_rel_ = V/V_norm_, as previously described [4]. Dogs with a S_rel_ > 1 thus had a larger prostate than would be normal for dogs 4 years or younger. S_rel_ ≥ 2.5 was considered a clinically relevant enlargement [4].

At baseline, the coefficient of variations (CVs) of the volume of the prostate within a dog, when measured by ultrasonography, was < 15% [4]. Based on this, volumes smaller or greater than 15% compared to measurements at baseline were defined as decreases or increases, respectively, and values in between were categorized as no significant change in volume.

A multiple regression analysis was applied to evaluate the effect of CPSE concentrations at baseline and at follow-up on S_rel_ at follow-up and on the difference between S_rel_ at baseline and at follow-up. Pearson correlation was used to evaluate the correlation between the CPSE concentration at baseline and S_rel_ at follow-up. Multiple regression analysis was also used to evaluate the effect of steroid concentrations at follow-up on S_rel_ at follow-up and on the difference between S_rel_ at baseline and at follow-up. In addition, two separate models were built, one including the steroids as the classes androgens, estrogens, progestins and corticosteroids, and the other including the ratios between the steroid classes. For the multiple regression analyses the residuals were checked and found to be normally distributed, and the use of multiple regression thus deemed appropriate.

Concentrations of CPSE at baseline and follow-up, as well as volume and relative size of the prostate, were mostly not normally distributed (evaluated using the Ryan-Joiner test), and values are therefore presented as median and interquartile range, IQR. Minitab (Minitab 18, Minitab Inc., State College, PA, USA) was used for comparing V and S_rel_ between baseline and follow-up, after numbers had been log-transformed, using the paired t-test. For comparison between groups with increased or similar prostate size between baseline and follow-up, the Kruskal-Wallis test was used. Fisher’s exact test was used to compare development of clinical signs depending on CPSE concentration or S_rel_ at baseline. McNemar’s test was used to compare the numbers of dogs with cysts at baseline and at follow-up. For the multiple regressions, R [39] was employed.

## Supplementary Information

**Additional file 1.** Follow-up contact (via phone) with owners of dogs included in a previous study. The file contains questions put forward to dog owners during an interview by telephone.

## Data Availability

The datasets used and/or analysed during the current study are available from the corresponding author on reasonable request.

## Abbreviations

BEH: Ethylene-bridged hybrid
BPH: Benign prostatic hyperplasia
CPSE: Canine prostate specific esterase
CV: Coefficient of variation
ER: Estrogen receptor
IQR: Interquartile range
MeOH: Methanol
MTBE: Methyl-tert-butyl ether
MRM: Multiple reaction monitoring
PSA: Prostate specific antigen
S_rel_: Relative prostatic size
SFC-MS/MS: Supercritical fluid chromatography – tandem mass spectrometry
V: Calculated prostatic volume
V_norm_: Normal prostatic volume

## Footnotes

1 GE Medical LOGIQ 9 Ultrasound Imaging System, General Electric Medical Systems, MyLab™ Alpha, Esaote SpA, *Philips IE33 Ultrasound, Philips Medical Systems*
