# Supplementary material for: Concentrations of canine prostate specific esterase, CPSE, at baseline are associated with the relative size of the prostate at three-year follow-up
Source: BMC Vet Res. 2021 Apr 26;17:173. doi: 10.1186/s12917-021-02874-1 (PMC8074475; doi:10.1186/s12917-021-02874-1)
Supplement: Supplementary file 1 — Additional file 1. Follow-up contact (via phone) with owners of dogs included in a previous study. The file contains questions put forward to dog owners during an interview by telephone. [file 12917_2021_2874_MOESM1_ESM.pdf]

Follow-up contact (via phone) with owners of dogs included in a previous study (1).

**Name of dog:**
**Breed:**
**Age:**
**Name of owner:**
**Telephone number:**
**E-mail address:**
**1. Is the dog still alive?**
If no, which was the cause of death?
**2. Since the previous sampling, has the dog developed clinical signs possibly related to prostatic disease?**
If yes, which signs?
**3. Has the dog been treated for prostatic disease?**
If yes,
Which diagnosis was made?
When was the dog treated?
Which treatment was given?
**4. Has the dog been castrated?**
If yes,
Why?
Was it a surgical or a medical castration?
**5. Does the dog have another disease?**
If yes, which:
**6. Do you want to participate in the study?**
If no, why not?

1. Holst BS, Holmroos E, Friling L, Hanås S, Langborg LM, Franko MA, Hansson K. The association between the serum concentration of canine prostate specific esterase (CPSE) and the size of the canine prostate. Theriogenology. 2017 Apr 15;93:33-39. doi: 10.1016/j.theriogenology.2017.01.032.
